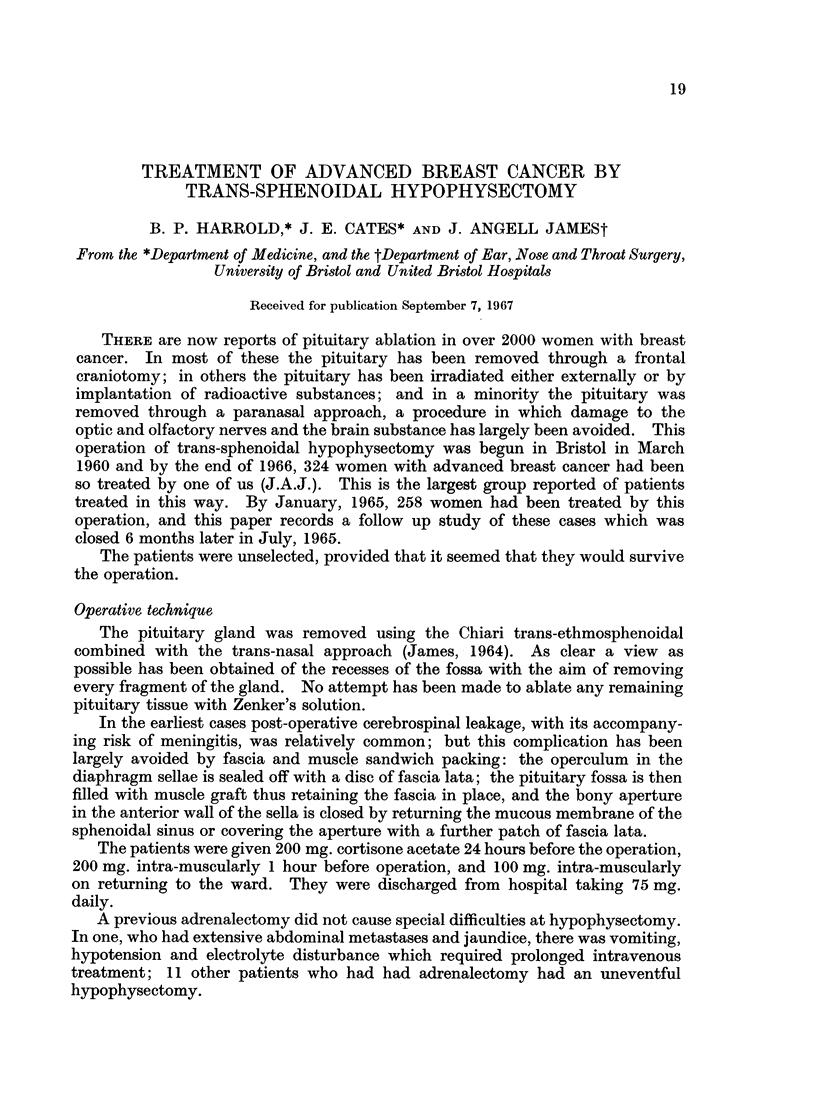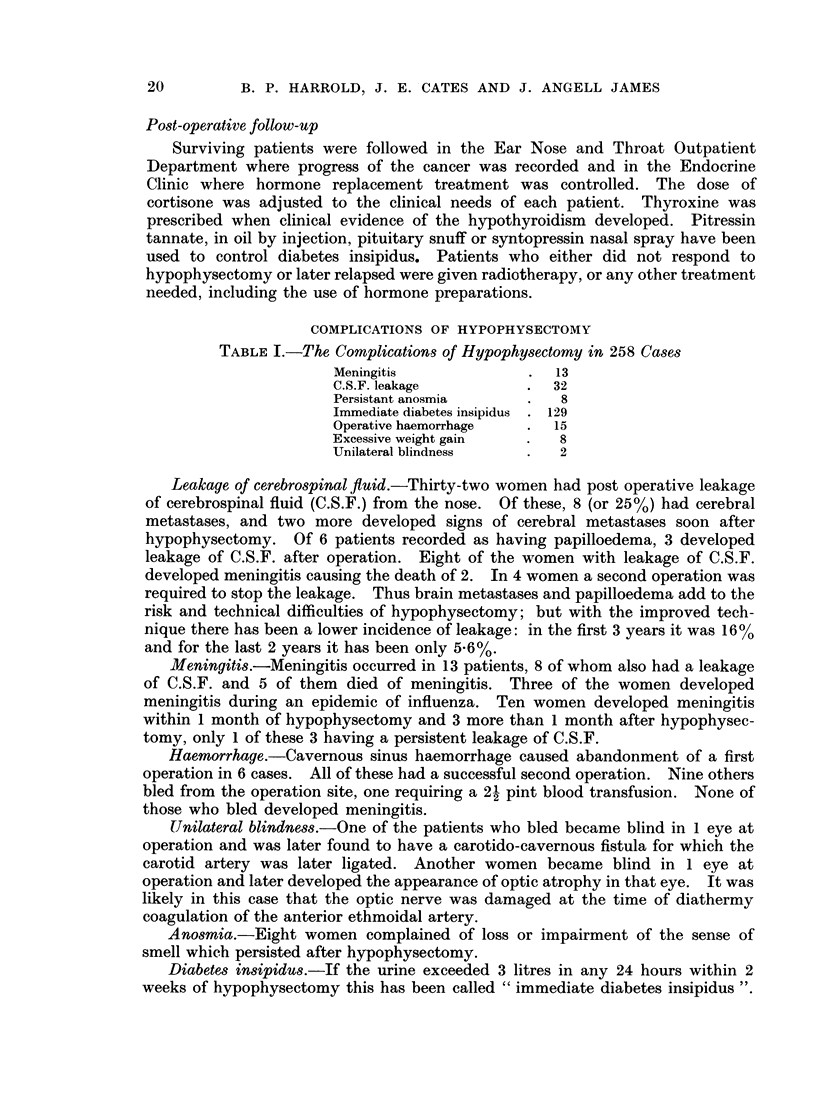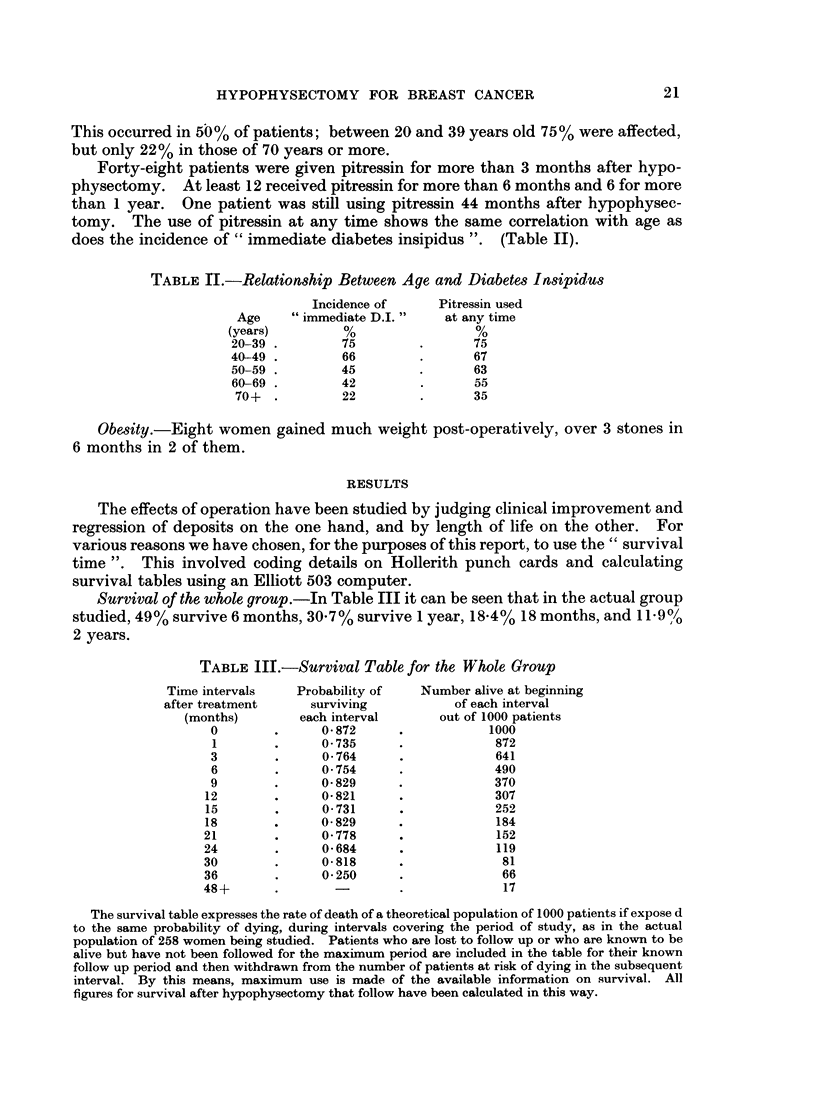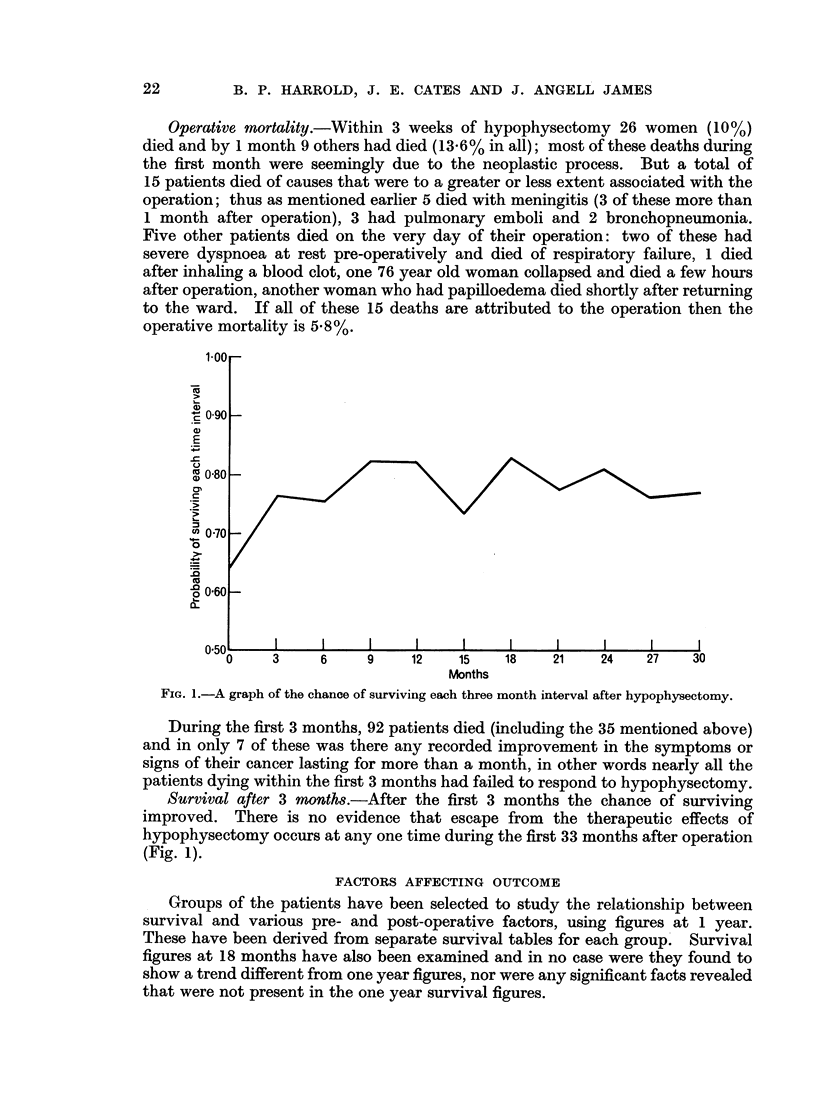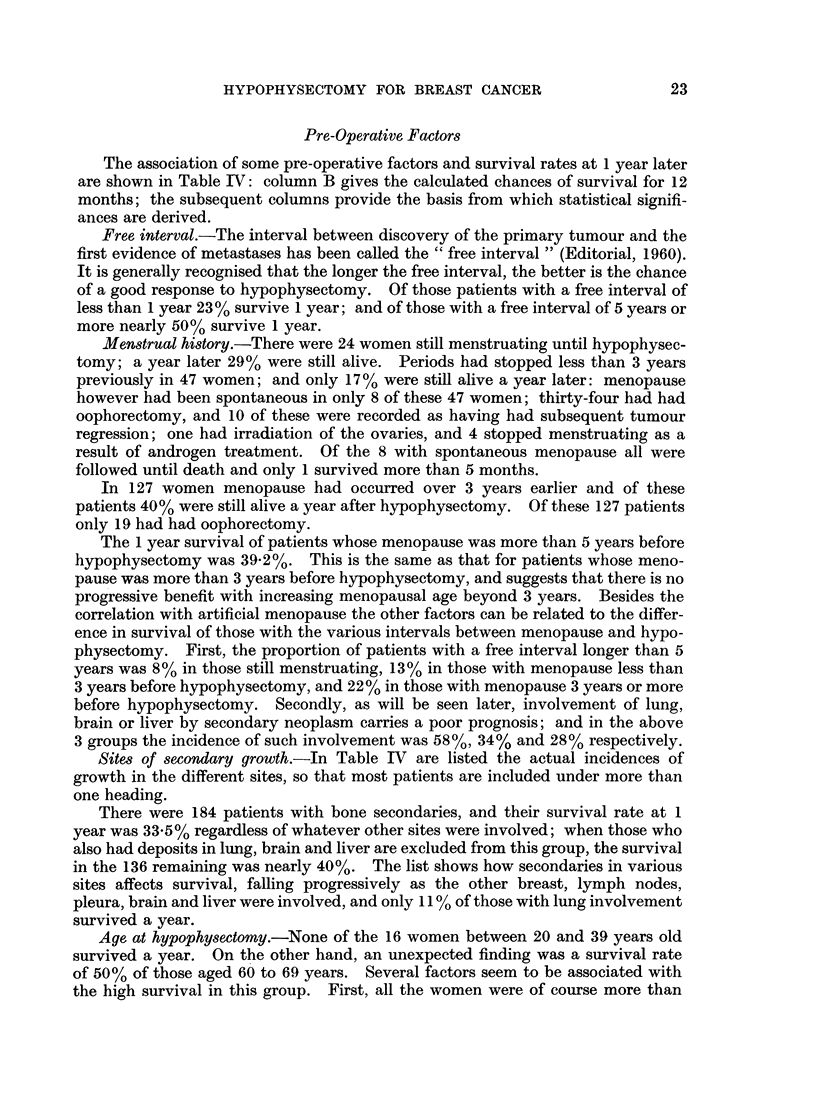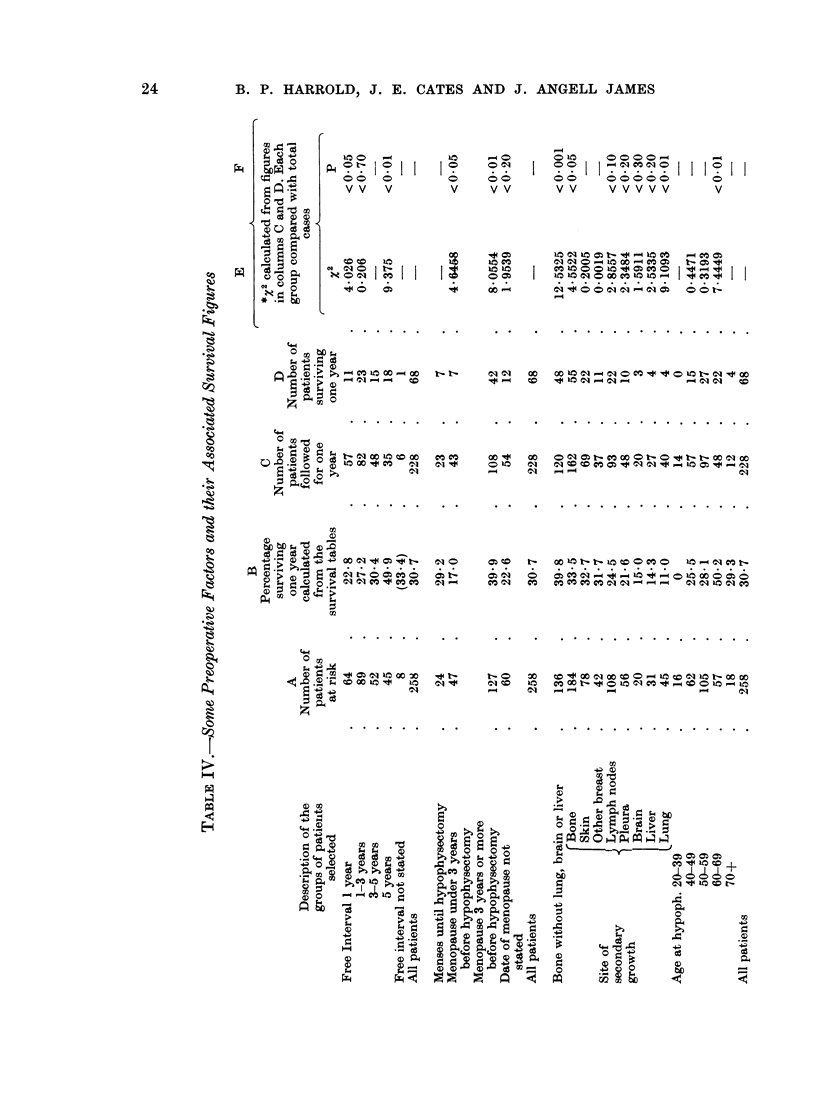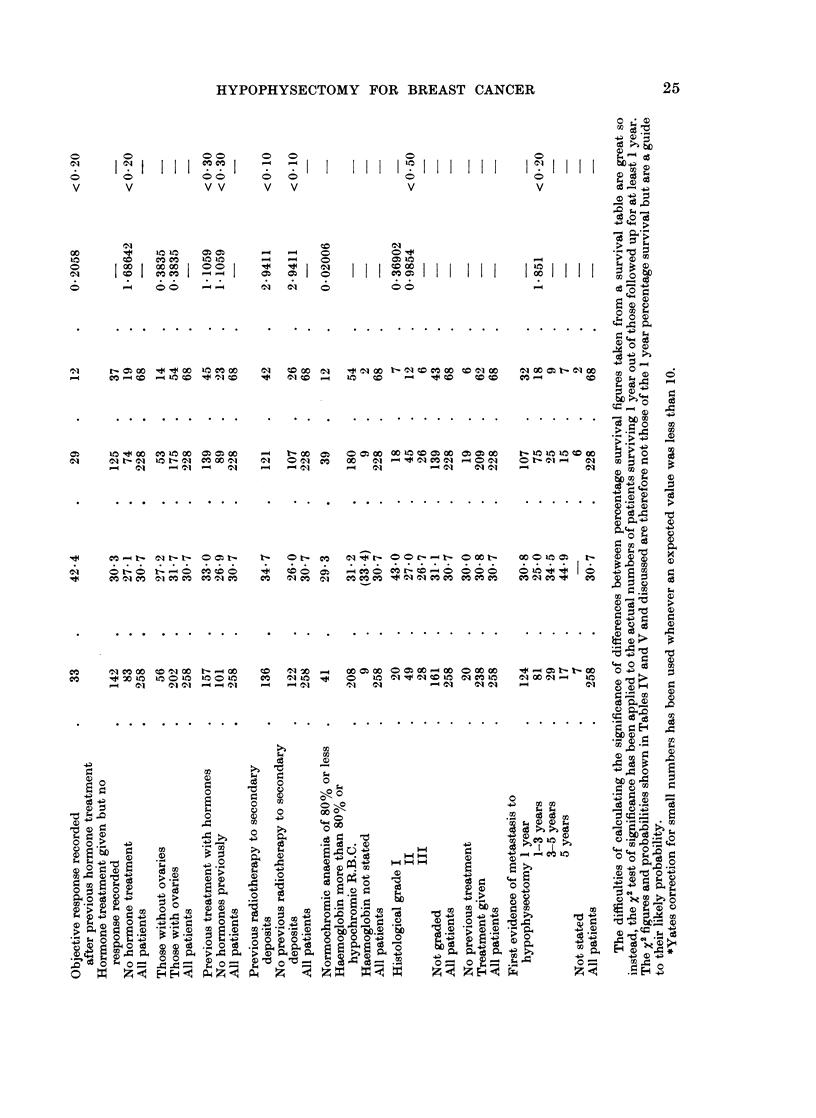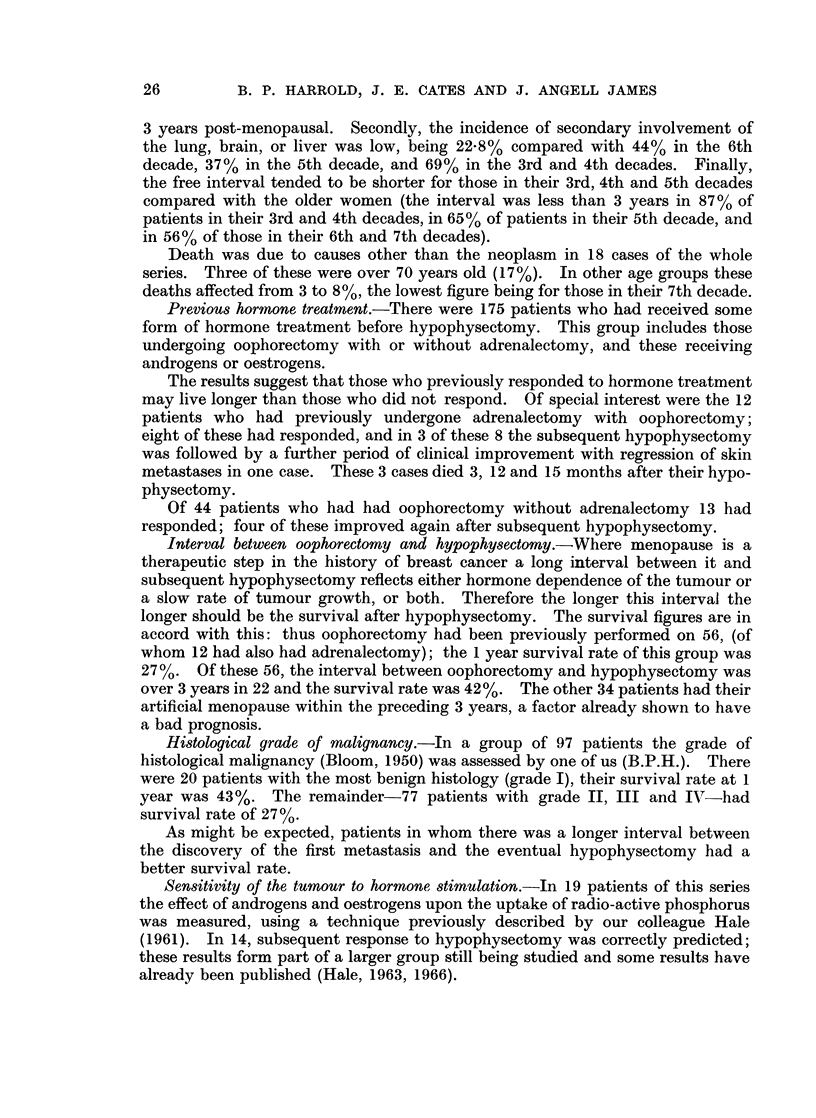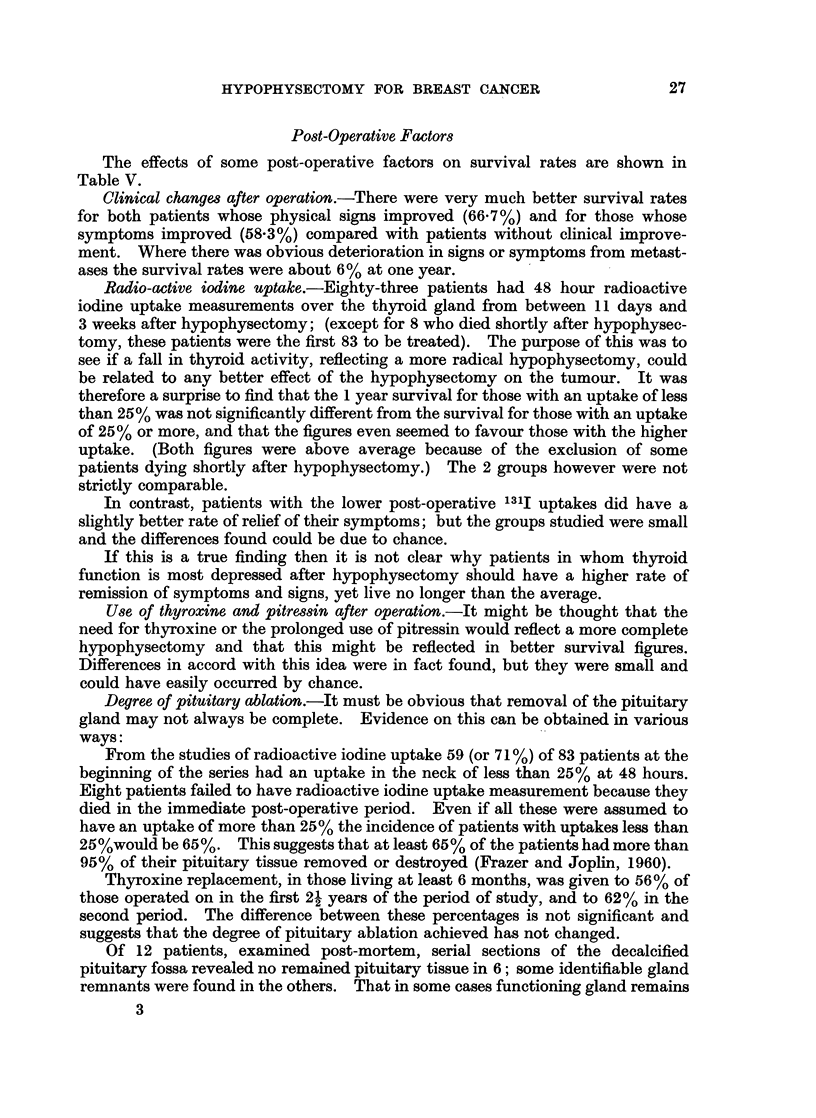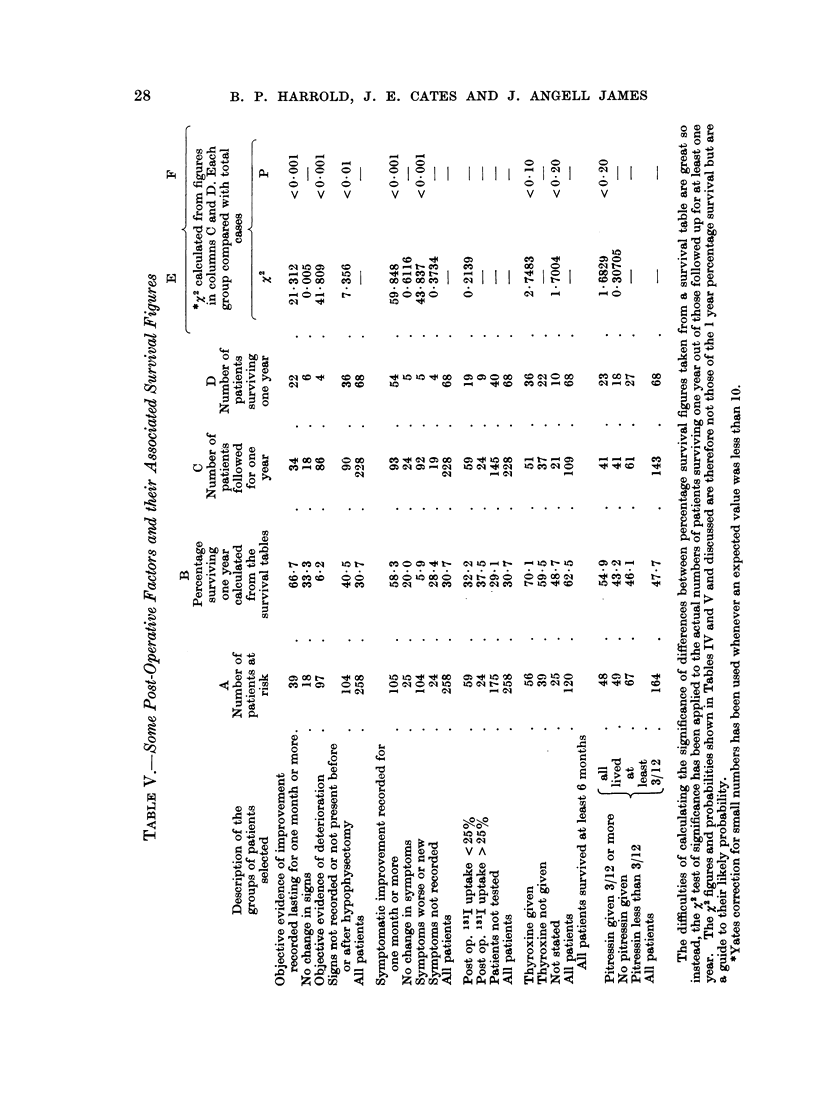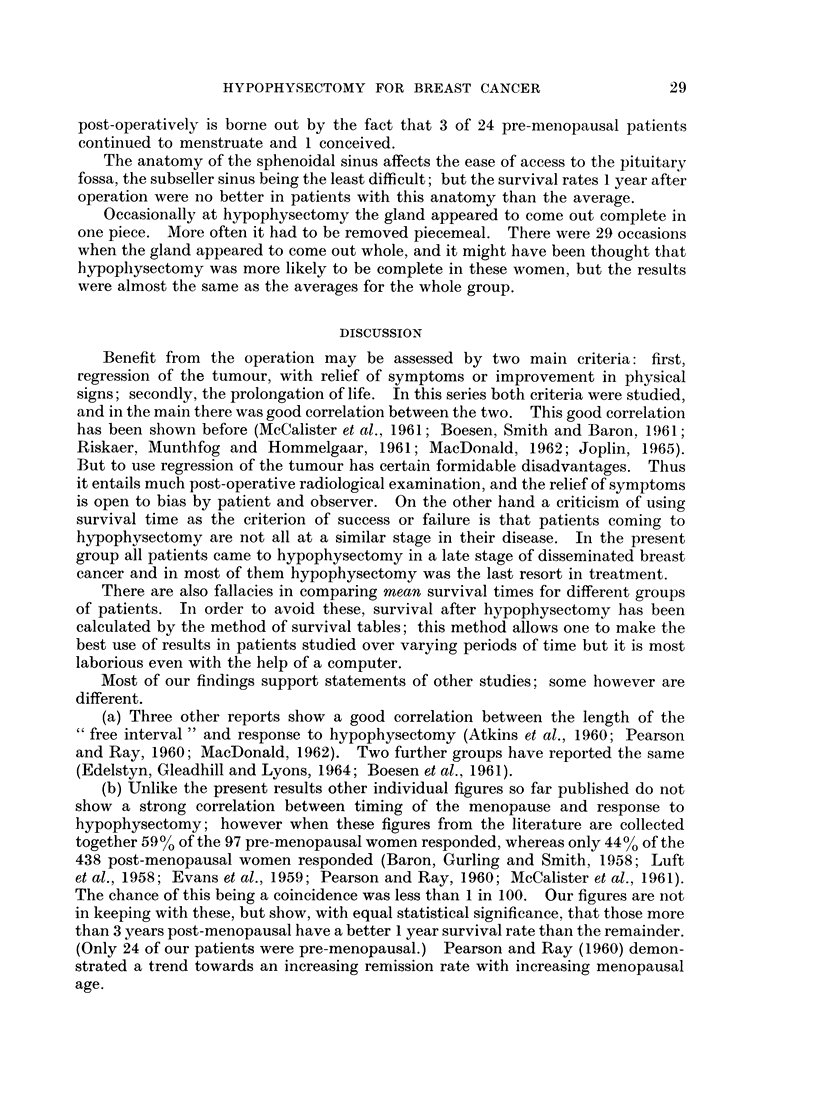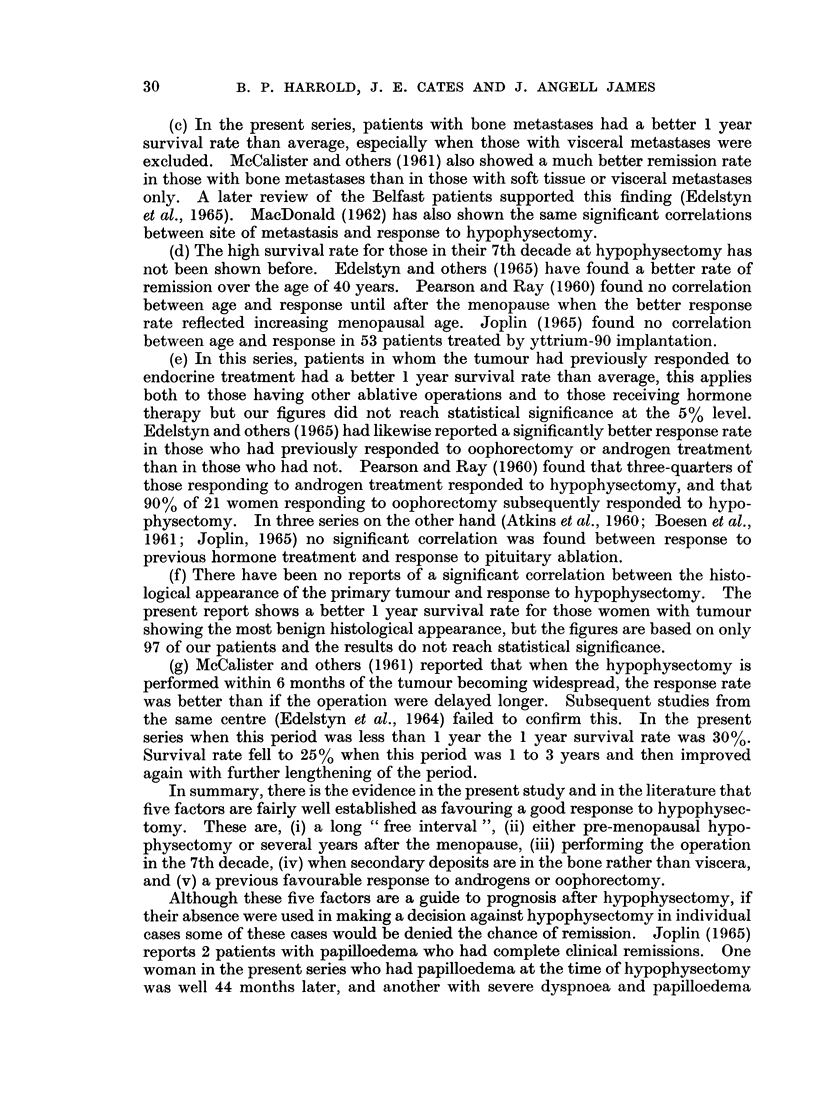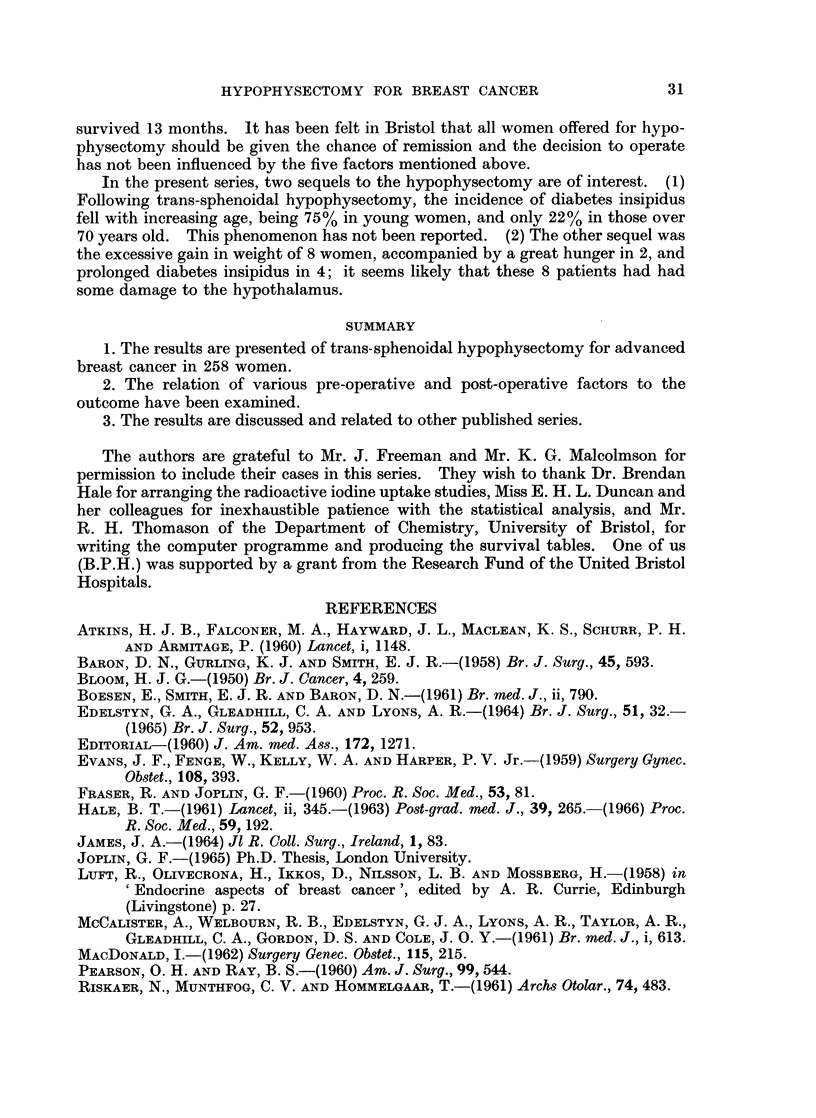# Treatment of advanced breast cancer by trans-sphenoidal hypophysectomy.

**DOI:** 10.1038/bjc.1968.4

**Published:** 1968-03

**Authors:** B. P. Harrold, J. E. Cates, J. A. James


### TREATMENT OF ADVANCED BREAST CANCER BY TRANS-SPHENOIDAL HYPOPHYSECTOMY

B. P. HARROLD,* J. E. CATES* AND J. ANGELL JAMES†

*From the *Department of Medicine, and the †Department of Ear, Nose and Throat Surgery, University of Bristol and United Bristol Hospitals*

Received for publication September 7, 1967

THERE are now reports of pituitary ablation in over 2000 women with breast cancer. In most of these the pituitary has been removed through a frontal craniotomy; in others the pituitary has been irradiated either externally or by implantation of radioactive substances; and in a minority the pituitary was removed through a paranasal approach, a procedure in which damage to the optic and olfactory nerves and the brain substance has largely been avoided. This operation of trans-sphenoidal hypophysectomy was begun in Bristol in March 1960 and by the end of 1966, 324 women with advanced breast cancer had been so treated by one of us (J.A.J.). This is the largest group reported of patients treated in this way. By January, 1965, 258 women had been treated by this operation, and this paper records a follow up study of these cases which was closed 6 months later in July, 1965.

The patients were unselected, provided that it seemed that they would survive the operation.

*Operative technique*

The pituitary gland was removed using the Chiari trans-ethmosphenoidal combined with the trans-nasal approach (James, 1964). As clear a view as possible has been obtained of the recesses of the fossa with the aim of removing every fragment of the gland. No attempt has been made to ablate any remaining pituitary tissue with Zenker's solution.

In the earliest cases post-operative cerebrospinal leakage, with its accompanying risk of meningitis, was relatively common; but this complication has been largely avoided by fascia and muscle sandwich packing: the operculum in the diaphragm sellae is sealed off with a disc of fascia lata; the pituitary fossa is then filled with muscle graft thus retaining the fascia in place, and the bony aperture in the anterior wall of the sella is closed by returning the mucous membrane of the sphenoidal sinus or covering the aperture with a further patch of fascia lata.

The patients were given 200 mg. cortisone acetate 24 hours before the operation, 200 mg. intra-muscularly 1 hour before operation, and 100 mg. intra-muscularly on returning to the ward. They were discharged from hospital taking 75 mg. daily.

A previous adrenalectomy did not cause special difficulties at hypophysectomy. In one, who had extensive abdominal metastases and jaundice, there was vomiting, hypotension and electrolyte disturbance which required prolonged intravenous treatment; 11 other patients who had had adrenalectomy had an uneventful hypophysectomy.

*Post-operative follow-up*

Surviving patients were followed in the Ear Nose and Throat Outpatient Department where progress of the cancer was recorded and in the Endocrine Clinic where hormone replacement treatment was controlled. The dose of cortisone was adjusted to the clinical needs of each patient. Thyroxine was prescribed when clinical evidence of the hypothyroidism developed. Pitressin tannate, in oil by injection, pituitary snuff or syntopressin nasal spray have been used to control diabetes insipidus. Patients who either did not respond to hypophysectomy or later relapsed were given radiotherapy, or any other treatment needed, including the use of hormone preparations.

### COMPLICATIONS OF HYPOPHYSECTOMY

TABLE I.—*The Complications of Hypophysectomy in* 258 *Cases*

| | |
|---|---|
| Meningitis | 13 |
| C.S.F. leakage | 32 |
| Persistant anosmia | 8 |
| Immediate diabetes insipidus | 129 |
| Operative haemorrhage | 15 |
| Excessive weight gain | 8 |
| Unilateral blindness | 2 |

*Leakage of cerebrospinal fluid.*—Thirty-two women had post operative leakage of cerebrospinal fluid (C.S.F.) from the nose. Of these, 8 (or 25%) had cerebral metastases, and two more developed signs of cerebral metastases soon after hypophysectomy. Of 6 patients recorded as having papilloedema, 3 developed leakage of C.S.F. after operation. Eight of the women with leakage of C.S.F. developed meningitis causing the death of 2. In 4 women a second operation was required to stop the leakage. Thus brain metastases and papilloedema add to the risk and technical difficulties of hypophysectomy; but with the improved technique there has been a lower incidence of leakage: in the first 3 years it was 16% and for the last 2 years it has been only 5·6%.

*Meningitis.*—Meningitis occurred in 13 patients, 8 of whom also had a leakage of C.S.F. and 5 of them died of meningitis. Three of the women developed meningitis during an epidemic of influenza. Ten women developed meningitis within 1 month of hypophysectomy and 3 more than 1 month after hypophysectomy, only 1 of these 3 having a persistent leakage of C.S.F.

*Haemorrhage.*—Cavernous sinus haemorrhage caused abandonment of a first operation in 6 cases. All of these had a successful second operation. Nine others bled from the operation site, one requiring a 2½ pint blood transfusion. None of those who bled developed meningitis.

*Unilateral blindness.*—One of the patients who bled became blind in 1 eye at operation and was later found to have a carotido-cavernous fistula for which the carotid artery was later ligated. Another women became blind in 1 eye at operation and later developed the appearance of optic atrophy in that eye. It was likely in this case that the optic nerve was damaged at the time of diathermy coagulation of the anterior ethmoidal artery.

*Anosmia.*—Eight women complained of loss or impairment of the sense of smell which persisted after hypophysectomy.

*Diabetes insipidus.*—If the urine exceeded 3 litres in any 24 hours within 2 weeks of hypophysectomy this has been called " immediate diabetes insipidus ".

This occurred in 50% of patients; between 20 and 39 years old 75% were affected, but only 22% in those of 70 years or more.

Forty-eight patients were given pitressin for more than 3 months after hypophysectomy. At least 12 received pitressin for more than 6 months and 6 for more than 1 year. One patient was still using pitressin 44 months after hypophysectomy. The use of pitressin at any time shows the same correlation with age as does the incidence of " immediate diabetes insipidus ". (Table II).

TABLE II.—*Relationship Between Age and Diabetes Insipidus*

| Age (years) | Incidence of " immediate D.I. " % | Pitressin used at any time % |
|---|---|---|
| 20–39 | 75 | 75 |
| 40–49 | 66 | 67 |
| 50–59 | 45 | 63 |
| 60–69 | 42 | 55 |
| 70+ | 22 | 35 |

*Obesity.*—Eight women gained much weight post-operatively, over 3 stones in 6 months in 2 of them.

### RESULTS

The effects of operation have been studied by judging clinical improvement and regression of deposits on the one hand, and by length of life on the other. For various reasons we have chosen, for the purposes of this report, to use the " survival time ". This involved coding details on Hollerith punch cards and calculating survival tables using an Elliott 503 computer.

*Survival of the whole group.*—In Table III it can be seen that in the actual group studied, 49% survive 6 months, 30·7% survive 1 year, 18·4% 18 months, and 11·9% 2 years.

TABLE III.—*Survival Table for the Whole Group*

| Time intervals after treatment (months) | Probability of surviving each interval | Number alive at beginning of each interval out of 1000 patients |
|---|---|---|
| 0 | 0·872 | 1000 |
| 1 | 0·735 | 872 |
| 3 | 0·764 | 641 |
| 6 | 0·754 | 490 |
| 9 | 0·829 | 370 |
| 12 | 0·821 | 307 |
| 15 | 0·731 | 252 |
| 18 | 0·829 | 184 |
| 21 | 0·778 | 152 |
| 24 | 0·684 | 119 |
| 30 | 0·818 | 81 |
| 36 | 0·250 | 66 |
| 48+ | — | 17 |

The survival table expresses the rate of death of a theoretical population of 1000 patients if expose d to the same probability of dying, during intervals covering the period of study, as in the actual population of 258 women being studied. Patients who are lost to follow up or who are known to be alive but have not been followed for the maximum period are included in the table for their known follow up period and then withdrawn from the number of patients at risk of dying in the subsequent interval. By this means, maximum use is made of the available information on survival. All figures for survival after hypophysectomy that follow have been calculated in this way.

*Operative mortality.*—Within 3 weeks of hypophysectomy 26 women (10%) died and by 1 month 9 others had died (13·6% in all); most of these deaths during the first month were seemingly due to the neoplastic process. But a total of 15 patients died of causes that were to a greater or less extent associated with the operation; thus as mentioned earlier 5 died with meningitis (3 of these more than 1 month after operation), 3 had pulmonary emboli and 2 bronchopneumonia. Five other patients died on the very day of their operation: two of these had severe dyspnoea at rest pre-operatively and died of respiratory failure, 1 died after inhaling a blood clot, one 76 year old woman collapsed and died a few hours after operation, another woman who had papilloedema died shortly after returning to the ward. If all of these 15 deaths are attributed to the operation then the operative mortality is 5·8%.

FIG. 1.—A graph of the chance of surviving each three month interval after hypophysectomy.

During the first 3 months, 92 patients died (including the 35 mentioned above) and in only 7 of these was there any recorded improvement in the symptoms or signs of their cancer lasting for more than a month, in other words nearly all the patients dying within the first 3 months had failed to respond to hypophysectomy.

*Survival after* 3 *months.*—After the first 3 months the chance of surviving improved. There is no evidence that escape from the therapeutic effects of hypophysectomy occurs at any one time during the first 33 months after operation (Fig. 1).

### FACTORS AFFECTING OUTCOME

Groups of the patients have been selected to study the relationship between survival and various pre- and post-operative factors, using figures at 1 year. These have been derived from separate survival tables for each group. Survival figures at 18 months have also been examined and in no case were they found to show a trend different from one year figures, nor were any significant facts revealed that were not present in the one year survival figures.

*Pre-Operative Factors*

The association of some pre-operative factors and survival rates at 1 year later are shown in Table IV: column B gives the calculated chances of survival for 12 months; the subsequent columns provide the basis from which statistical significances are derived.

*Free interval.*—The interval between discovery of the primary tumour and the first evidence of metastases has been called the " free interval " (Editorial, 1960). It is generally recognised that the longer the free interval, the better is the chance of a good response to hypophysectomy. Of those patients with a free interval of less than 1 year 23% survive 1 year; and of those with a free interval of 5 years or more nearly 50% survive 1 year.

*Menstrual history.*—There were 24 women still menstruating until hypophysectomy; a year later 29% were still alive. Periods had stopped less than 3 years previously in 47 women; and only 17% were still alive a year later: menopause however had been spontaneous in only 8 of these 47 women; thirty-four had had oophorectomy, and 10 of these were recorded as having had subsequent tumour regression; one had irradiation of the ovaries, and 4 stopped menstruating as a result of androgen treatment. Of the 8 with spontaneous menopause all were followed until death and only 1 survived more than 5 months.

In 127 women menopause had occurred over 3 years earlier and of these patients 40% were still alive a year after hypophysectomy. Of these 127 patients only 19 had had oophorectomy.

The 1 year survival of patients whose menopause was more than 5 years before hypophysectomy was 39·2%. This is the same as that for patients whose menopause was more than 3 years before hypophysectomy, and suggests that there is no progressive benefit with increasing menopausal age beyond 3 years. Besides the correlation with artificial menopause the other factors can be related to the difference in survival of those with the various intervals between menopause and hypophysectomy. First, the proportion of patients with a free interval longer than 5 years was 8% in those still menstruating, 13% in those with menopause less than 3 years before hypophysectomy, and 22% in those with menopause 3 years or more before hypophysectomy. Secondly, as will be seen later, involvement of lung, brain or liver by secondary neoplasm carries a poor prognosis; and in the above 3 groups the incidence of such involvement was 58%, 34% and 28% respectively.

*Sites of secondary growth.*—In Table IV are listed the actual incidences of growth in the different sites, so that most patients are included under more than one heading.

There were 184 patients with bone secondaries, and their survival rate at 1 year was 33·5% regardless of whatever other sites were involved; when those who also had deposits in lung, brain and liver are excluded from this group, the survival in the 136 remaining was nearly 40%. The list shows how secondaries in various sites affects survival, falling progressively as the other breast, lymph nodes, pleura, brain and liver were involved, and only 11% of those with lung involvement survived a year.

*Age at hypophysectomy.*—None of the 16 women between 20 and 39 years old survived a year. On the other hand, an unexpected finding was a survival rate of 50% of those aged 60 to 69 years. Several factors seem to be associated with the high survival in this group. First, all the women were of course more than

3 years post-menopausal. Secondly, the incidence of secondary involvement of the lung, brain, or liver was low, being 22·8% compared with 44% in the 6th decade, 37% in the 5th decade, and 69% in the 3rd and 4th decades. Finally, the free interval tended to be shorter for those in their 3rd, 4th and 5th decades compared with the older women (the interval was less than 3 years in 87% of patients in their 3rd and 4th decades, in 65% of patients in their 5th decade, and in 56% of those in their 6th and 7th decades).

Death was due to causes other than the neoplasm in 18 cases of the whole series. Three of these were over 70 years old (17%). In other age groups these deaths affected from 3 to 8%, the lowest figure being for those in their 7th decade.

*Previous hormone treatment.*—There were 175 patients who had received some form of hormone treatment before hypophysectomy. This group includes those undergoing oophorectomy with or without adrenalectomy, and these receiving androgens or oestrogens.

The results suggest that those who previously responded to hormone treatment may live longer than those who did not respond. Of special interest were the 12 patients who had previously undergone adrenalectomy with oophorectomy; eight of these had responded, and in 3 of these 8 the subsequent hypophysectomy was followed by a further period of clinical improvement with regression of skin metastases in one case. These 3 cases died 3, 12 and 15 months after their hypophysectomy.

Of 44 patients who had had oophorectomy without adrenalectomy 13 had responded; four of these improved again after subsequent hypophysectomy.

*Interval between oophorectomy and hypophysectomy.*—Where menopause is a therapeutic step in the history of breast cancer a long interval between it and subsequent hypophysectomy reflects either hormone dependence of the tumour or a slow rate of tumour growth, or both. Therefore the longer this interval the longer should be the survival after hypophysectomy. The survival figures are in accord with this: thus oophorectomy had been previously performed on 56, (of whom 12 had also had adrenalectomy); the 1 year survival rate of this group was 27%. Of these 56, the interval between oophorectomy and hypophysectomy was over 3 years in 22 and the survival rate was 42%. The other 34 patients had their artificial menopause within the preceding 3 years, a factor already shown to have a bad prognosis.

*Histological grade of malignancy.*—In a group of 97 patients the grade of histological malignancy (Bloom, 1950) was assessed by one of us (B.P.H.). There were 20 patients with the most benign histology (grade I), their survival rate at 1 year was 43%. The remainder—77 patients with grade II, III and IV—had survival rate of 27%.

As might be expected, patients in whom there was a longer interval between the discovery of the first metastasis and the eventual hypophysectomy had a better survival rate.

*Sensitivity of the tumour to hormone stimulation.*—In 19 patients of this series the effect of androgens and oestrogens upon the uptake of radio-active phosphorus was measured, using a technique previously described by our colleague Hale (1961). In 14, subsequent response to hypophysectomy was correctly predicted; these results form part of a larger group still being studied and some results have already been published (Hale, 1963, 1966).

*Post-Operative Factors*

The effects of some post-operative factors on survival rates are shown in Table V.

*Clinical changes after operation.*—There were very much better survival rates for both patients whose physical signs improved (66·7%) and for those whose symptoms improved (58·3%) compared with patients without clinical improvement. Where there was obvious deterioration in signs or symptoms from metastases the survival rates were about 6% at one year.

*Radio-active iodine uptake.*—Eighty-three patients had 48 hour radioactive iodine uptake measurements over the thyroid gland from between 11 days and 3 weeks after hypophysectomy; (except for 8 who died shortly after hypophysectomy, these patients were the first 83 to be treated). The purpose of this was to see if a fall in thyroid activity, reflecting a more radical hypophysectomy, could be related to any better effect of the hypophysectomy on the tumour. It was therefore a surprise to find that the 1 year survival for those with an uptake of less than 25% was not significantly different from the survival for those with an uptake of 25% or more, and that the figures even seemed to favour those with the higher uptake. (Both figures were above average because of the exclusion of some patients dying shortly after hypophysectomy.) The 2 groups however were not strictly comparable.

In contrast, patients with the lower post-operative $^{131}$I uptakes did have a slightly better rate of relief of their symptoms; but the groups studied were small and the differences found could be due to chance.

If this is a true finding then it is not clear why patients in whom thyroid function is most depressed after hypophysectomy should have a higher rate of remission of symptoms and signs, yet live no longer than the average.

*Use of thyroxine and pitressin after operation.*—It might be thought that the need for thyroxine or the prolonged use of pitressin would reflect a more complete hypophysectomy and that this might be reflected in better survival figures. Differences in accord with this idea were in fact found, but they were small and could have easily occurred by chance.

*Degree of pituitary ablation.*—It must be obvious that removal of the pituitary gland may not always be complete. Evidence on this can be obtained in various ways:

From the studies of radioactive iodine uptake 59 (or 71%) of 83 patients at the beginning of the series had an uptake in the neck of less than 25% at 48 hours. Eight patients failed to have radioactive iodine uptake measurement because they died in the immediate post-operative period. Even if all these were assumed to have an uptake of more than 25% the incidence of patients with uptakes less than 25%would be 65%. This suggests that at least 65% of the patients had more than 95% of their pituitary tissue removed or destroyed (Frazer and Joplin, 1960).

Thyroxine replacement, in those living at least 6 months, was given to 56% of those operated on in the first 2½ years of the period of study, and to 62% in the second period. The difference between these percentages is not significant and suggests that the degree of pituitary ablation achieved has not changed.

Of 12 patients, examined post-mortem, serial sections of the decalcified pituitary fossa revealed no remained pituitary tissue in 6; some identifiable gland remnants were found in the others. That in some cases functioning gland remains post-operatively is borne out by the fact that 3 of 24 pre-menopausal patients continued to menstruate and 1 conceived.

The anatomy of the sphenoidal sinus affects the ease of access to the pituitary fossa, the subseller sinus being the least difficult; but the survival rates 1 year after operation were no better in patients with this anatomy than the average.

Occasionally at hypophysectomy the gland appeared to come out complete in one piece. More often it had to be removed piecemeal. There were 29 occasions when the gland appeared to come out whole, and it might have been thought that hypophysectomy was more likely to be complete in these women, but the results were almost the same as the averages for the whole group.

### DISCUSSION

Benefit from the operation may be assessed by two main criteria: first, regression of the tumour, with relief of symptoms or improvement in physical signs; secondly, the prolongation of life. In this series both criteria were studied, and in the main there was good correlation between the two. This good correlation has been shown before (McCalister *et al.*, 1961; Boesen, Smith and Baron, 1961; Riskaer, Munthfog and Hommelgaar, 1961; MacDonald, 1962; Joplin, 1965). But to use regression of the tumour has certain formidable disadvantages. Thus it entails much post-operative radiological examination, and the relief of symptoms is open to bias by patient and observer. On the other hand a criticism of using survival time as the criterion of success or failure is that patients coming to hypophysectomy are not all at a similar stage in their disease. In the present group all patients came to hypophysectomy in a late stage of disseminated breast cancer and in most of them hypophysectomy was the last resort in treatment.

There are also fallacies in comparing *mean* survival times for different groups of patients. In order to avoid these, survival after hypophysectomy has been calculated by the method of survival tables; this method allows one to make the best use of results in patients studied over varying periods of time but it is most laborious even with the help of a computer.

Most of our findings support statements of other studies; some however are different.

(a) Three other reports show a good correlation between the length of the " free interval " and response to hypophysectomy (Atkins *et al.*, 1960; Pearson and Ray, 1960; MacDonald, 1962). Two further groups have reported the same (Edelstyn, Gleadhill and Lyons, 1964; Boesen *et al.*, 1961).

(b) Unlike the present results other individual figures so far published do not show a strong correlation between timing of the menopause and response to hypophysectomy; however when these figures from the literature are collected together 59% of the 97 pre-menopausal women responded, whereas only 44% of the 438 post-menopausal women responded (Baron, Gurling and Smith, 1958; Luft *et al.*, 1958; Evans *et al.*, 1959; Pearson and Ray, 1960; McCalister *et al.*, 1961). The chance of this being a coincidence was less than 1 in 100. Our figures are not in keeping with these, but show, with equal statistical significance, that those more than 3 years post-menopausal have a better 1 year survival rate than the remainder. (Only 24 of our patients were pre-menopausal.) Pearson and Ray (1960) demonstrated a trend towards an increasing remission rate with increasing menopausal age.

(c) In the present series, patients with bone metastases had a better 1 year survival rate than average, especially when those with visceral metastases were excluded. McCalister and others (1961) also showed a much better remission rate in those with bone metastases than in those with soft tissue or visceral metastases only. A later review of the Belfast patients supported this finding (Edelstyn *et al.*, 1965). MacDonald (1962) has also shown the same significant correlations between site of metastasis and response to hypophysectomy.

(d) The high survival rate for those in their 7th decade at hypophysectomy has not been shown before. Edelstyn and others (1965) have found a better rate of remission over the age of 40 years. Pearson and Ray (1960) found no correlation between age and response until after the menopause when the better response rate reflected increasing menopausal age. Joplin (1965) found no correlation between age and response in 53 patients treated by yttrium-90 implantation.

(e) In this series, patients in whom the tumour had previously responded to endocrine treatment had a better 1 year survival rate than average, this applies both to those having other ablative operations and to those receiving hormone therapy but our figures did not reach statistical significance at the 5% level. Edelstyn and others (1965) had likewise reported a significantly better response rate in those who had previously responded to oophorectomy or androgen treatment than in those who had not. Pearson and Ray (1960) found that three-quarters of those responding to androgen treatment responded to hypophysectomy, and that 90% of 21 women responding to oophorectomy subsequently responded to hypophysectomy. In three series on the other hand (Atkins *et al.*, 1960; Boesen *et al.*, 1961; Joplin, 1965) no significant correlation was found between response to previous hormone treatment and response to pituitary ablation.

(f) There have been no reports of a significant correlation between the histological appearance of the primary tumour and response to hypophysectomy. The present report shows a better 1 year survival rate for those women with tumour showing the most benign histological appearance, but the figures are based on only 97 of our patients and the results do not reach statistical significance.

(g) McCalister and others (1961) reported that when the hypophysectomy is performed within 6 months of the tumour becoming widespread, the response rate was better than if the operation were delayed longer. Subsequent studies from the same centre (Edelstyn *et al.*, 1964) failed to confirm this. In the present series when this period was less than 1 year the 1 year survival rate was 30%. Survival rate fell to 25% when this period was 1 to 3 years and then improved again with further lengthening of the period.

In summary, there is the evidence in the present study and in the literature that five factors are fairly well established as favouring a good response to hypophysectomy. These are, (i) a long " free interval ", (ii) either pre-menopausal hypophysectomy or several years after the menopause, (iii) performing the operation in the 7th decade, (iv) when secondary deposits are in the bone rather than viscera, and (v) a previous favourable response to androgens or oophorectomy.

Although these five factors are a guide to prognosis after hypophysectomy, if their absence were used in making a decision against hypophysectomy in individual cases some of these cases would be denied the chance of remission. Joplin (1965) reports 2 patients with papilloedema who had complete clinical remissions. One woman in the present series who had papilloedema at the time of hypophysectomy was well 44 months later, and another with severe dyspnoea and papilloedema survived 13 months. It has been felt in Bristol that all women offered for hypophysectomy should be given the chance of remission and the decision to operate has not been influenced by the five factors mentioned above.

In the present series, two sequels to the hypophysectomy are of interest. (1) Following trans-sphenoidal hypophysectomy, the incidence of diabetes insipidus fell with increasing age, being 75% in young women, and only 22% in those over 70 years old. This phenomenon has not been reported. (2) The other sequel was the excessive gain in weight of 8 women, accompanied by a great hunger in 2, and prolonged diabetes insipidus in 4; it seems likely that these 8 patients had had some damage to the hypothalamus.

### SUMMARY

1. The results are presented of trans-sphenoidal hypophysectomy for advanced breast cancer in 258 women.

2. The relation of various pre-operative and post-operative factors to the outcome have been examined.

3. The results are discussed and related to other published series.

The authors are grateful to Mr. J. Freeman and Mr. K. G. Malcolmson for permission to include their cases in this series. They wish to thank Dr. Brendan Hale for arranging the radioactive iodine uptake studies, Miss E. H. L. Duncan and her colleagues for inexhaustible patience with the statistical analysis, and Mr. R. H. Thomason of the Department of Chemistry, University of Bristol, for writing the computer programme and producing the survival tables. One of us (B.P.H.) was supported by a grant from the Research Fund of the United Bristol Hospitals.

### REFERENCES

Atkins, H. J. B., Falconer, M. A., Hayward, J. L., Maclean, K. S., Schurr, P. H. and Armitage, P. (1960) *Lancet*, i, 1148.

Baron, D. N., Gurling, K. J. and Smith, E. J. R.—(1958) *Br. J. Surg.*, **45**, 593.

Bloom, H. J. G.—(1950) *Br. J. Cancer*, **4**, 259.

Boesen, E., Smith, E. J. R. and Baron, D. N.—(1961) *Br. med. J.*, ii, 790.

Edelstyn, G. A., Gleadhill, C. A. and Lyons, A. R.—(1964) *Br. J. Surg.*, **51**, 32.—(1965) *Br. J. Surg.*, **52**, 953.

Editorial—(1960) *J. Am. med. Ass.*, **172**, 1271.

Evans, J. F., Fenge, W., Kelly, W. A. and Harper, P. V. Jr.—(1959) *Surgery Gynec. Obstet.*, **108**, 393.

Fraser, R. and Joplin, G. F.—(1960) *Proc. R. Soc. Med.*, **53**, 81.

Hale, B. T.—(1961) *Lancet*, ii, 345.—(1963) *Post-grad. med. J.*, **39**, 265.—(1966) *Proc. R. Soc. Med.*, **59**, 192.

James, J. A.—(1964) *Jl R. Coll. Surg., Ireland*, **1**, 83.

Joplin, G. F.—(1965) Ph.D. Thesis, London University.

Luft, R., Olivecrona, H., Ikkos, D., Nilsson, L. B. and Mossberg, H.—(1958) *in* ' Endocrine aspects of breast cancer ', edited by A. R. Currie, Edinburgh (Livingstone) p. 27.

McCalister, A., Welbourn, R. B., Edelstyn, G. J. A., Lyons, A. R., Taylor, A. R., Gleadhill, C. A., Gordon, D. S. and Cole, J. O. Y.—(1961) *Br. med. J.*, i, 613.

MacDonald, I.—(1962) *Surgery Genec. Obstet.*, **115**, 215.

Pearson, O. H. and Ray, B. S.—(1960) *Am. J. Surg.*, **99**, 544.

Riskaer, N., Munthfog, C. V. and Hommelgaar, T.—(1961) *Archs Otolar.*, **74**, 483.